# Quality of Sleep Data Validation From the Xiaomi Mi Band 5 Against Polysomnography: Comparison Study

**DOI:** 10.2196/42073

**Published:** 2023-05-19

**Authors:** Patricia Concheiro-Moscoso, Betania Groba, Diego Alvarez-Estevez, María del Carmen Miranda-Duro, Thais Pousada, Laura Nieto-Riveiro, Francisco Javier Mejuto-Muiño, Javier Pereira

**Affiliations:** 1 CITIC-TALIONIS Group, Elviña Campus Universidade da Coruña (University of A Coruña) A Coruña Spain; 2 Faculty of Health Sciences, Oza Campus Universidade da Coruña (University of A Coruña) A Coruña Spain; 3 CITIC, Elviña Campus Universidade da Coruña (University of A Coruña) A Coruña Spain; 4 Clinical Neurophysiology Service Hospital San Rafael A Coruña Spain

**Keywords:** sleep, health promotion, occupation, polysomnography, Xiaomi Mi Band 5, Internet of Things

## Abstract

**Background:**

Polysomnography is the gold standard for measuring and detecting sleep patterns. In recent years, activity wristbands have become popular because they record continuous data in real time. Hence, comprehensive validation studies are needed to analyze the performance and reliability of these devices in the recording of sleep parameters.

**Objective:**

This study compared the performance of one of the best-selling activity wristbands, the Xiaomi Mi Band 5, against polysomnography in measuring sleep stages.

**Methods:**

This study was carried out at a hospital in A Coruña, Spain. People who were participating in a polysomnography study at a sleep unit were recruited to wear a Xiaomi Mi Band 5 simultaneously for 1 night. The total sample consisted of 45 adults, 25 (56%) with sleep disorders (SDis) and 20 (44%) without SDis.

**Results:**

Overall, the Xiaomi Mi Band 5 displayed 78% accuracy, 89% sensitivity, 35% specificity, and a Cohen κ value of 0.22. It significantly overestimated polysomnography total sleep time (*P*=.09), *light sleep* (N1+N2 stages of non–rapid eye movement [REM] sleep; *P*=.005), and *deep sleep* (N3 stage of non-REM sleep; *P*=.01). In addition, it underestimated polysomnography wake after sleep onset and REM sleep. Moreover, the Xiaomi Mi Band 5 performed better in people without sleep problems than in those with sleep problems, specifically in detecting total sleep time and *deep sleep*.

**Conclusions:**

The Xiaomi Mi Band 5 can be potentially used to monitor sleep and to detect changes in sleep patterns, especially for people without sleep problems. However, additional studies are necessary with this activity wristband in people with different types of SDis.

**Trial Registration:**

ClinicalTrials.gov NCT04568408; https://clinicaltrials.gov/ct2/show/NCT04568408

**International Registered Report Identifier (IRRID):**

RR2-10.3390/ijerph18031106

## Introduction

### Background

In recent years, technological advances have made it possible to carry out diverse daily tasks (eg, health management) more quickly, efficiently, and immediately [1,2]. In this context, the Internet of Things is revolutionizing the health care system, allowing daily users to monitor their health status in real time through, for example, wearable devices [2]. Thus, the Internet of Things has spurred a digital social transformation resulting from changes in lifestyle patterns through the connection between everyday life and communication networks [3].

Activity wristbands have become more popular among consumers because of their usefulness, affordability, and attractive design [4]. Sales of these device have risen in recent years, with approximately 65.1 million units sold worldwide [5]. The technology industry has moved to brand these devices as an increasingly rigorous option for measuring biomedical parameters [4]. Scientific evidence indicates that these wristbands can promote participatory medicine because they encourage people to be active agents in their health management [6]. Moreover, several studies indicate that these devices can help stakeholders to become more aware of their health status and to improve their healthy lifestyle habits [7,8].

Activity wristbands can collect a myriad of daily health information in the user’s free-living environments [9,10]. Although these devices were designed primarily to record physical activity, companies are increasingly focused on developing algorithms that record other variables such as sleep and its patterns [9,10]. Thus, the use of wristbands not only aims to monitor sleep patterns but also contributes to make the population more aware of sleep relevance [11,12]. In this sense, it is important to know the details of sleep behavior in the population because sleep is a vital part of daily life and a determinant of health and well-being [13-15].

Polysomnography (PSG) represents the reference method used to measure sleep patterns. Its objective is the diagnosis of sleep problems by assessing the quality and quantity of sleep [16,17]. However, PSG is limited to the clinical setting owing to its cost, invasiveness, the need for specialized professionals, and difficulty functioning in the user’s free-living environment [15,18]. Therefore, clinicians consider actigraphy to be a common alternative to address the drawbacks of PSG [19,20]. Several studies have compared actigraphy with PSG, showing that the former provides high sensitivity in detecting sleep epochs but low specificity in detecting wakefulness [20-22]. Actigraphy has poor accessibility, and a lack of feedback to users hinders its daily use in the general population [11,20]. Nevertheless, it is a validated and accepted tool for detecting certain sleep disorders (SDis) at the clinical and research levels [15,21].

On the basis of actigraphy, activity wristbands can combine movement signals from an accelerometer and heart rate (HR) variability from sensors to detect sleep-wake cycles [23,24]. Recently, some sleep researchers have considered these devices to be a potential complement to traditional sleep assessment methods because they can sense long-term variations in the circadian rhythm and sleep quantity and quality [4,25,26]. However, previous evidence indicates that the sleep data recorded by these devices are unreliable because they do not accurately detect sleep stages, being more precise in detecting sleep than wakefulness [9,19,27]. Hence, health professionals have stated that data from these devices can lead to excessive concern among consumers about getting optimal sleep, a phenomenon known as *orthosomnia* [28,29]. In addition, the lack of access to the raw data and algorithms used for sleep parameter measurements raises doubts about their use in clinical and research settings [4,27,30].

Given the aforementioned limitations, sleep health entities, such as the American Academy of Sleep Medicine (AASM), consider it necessary to perform validation studies of activity devices to evaluate their performance and reliability against PSG, which is the gold standard [4,31,32]. In this view, it is relevant that validation studies follow some criteria to assess these devices, such as the American National Standards Institute (ANSI) and Consumer Technology Association (CTA) standard [33]. This standard is based on 2 levels of compliance to test how the devices classify sleep and wake stages and how they classify sleep stages (wake, rapid eye movement [REM] sleep, *light sleep*, and *deep sleep*) [33].

Few studies have analyzed the sleep data performance of activity wristbands. Most of these studies focused on validation of the Fitbit [34-37], Jawbone UP [10,38], and Oura ring [39,40] devices. In general, compared with PSG, these devices have good accuracy in sleep versus wake differentiation (between 65% and 91%) and high sensitivity (between 96% and 97%) but low specificity (between 42% and 51%) [10,34-39]. These devices overestimate PSG total sleep time (TST) and sleep efficiency (SE), and they underestimate PSG wake after sleep onset (WASO) and sleep onset latency (SOL) [10,34-39,41,42].

Some of these validated devices can also identify sleep stages, although the Fitbit Charge 2 overestimates PSG N1+N2 stages of non-REM sleep and underestimates N3 stage of non-REM sleep [36,37], whereas the Oura ring overestimates PSG REM sleep and underestimates N3 sleep [39]. In addition, the authors of some comparative studies claim that it is essential to know the performance of these devices in people with SDis, considering that most studies have only included healthy populations [23,34,36]. Kahawage et al [34] and Moreno-Pino et al [36] used the Fitbit Alta HR in a population with obstructive sleep apnea (OSA) and insomnia. Both studies showed similar concordance against PSG, with high sensitivity and low specificity [34,36].

According to the literature, it is necessary to continue testing the validity of wearable devices [11,30]. Therefore, this research focused on the Xiaomi Mi Band (Xiaomi Inc), which is one of the most popular activity wristbands on the market owing to its low cost and suitable quality [4,5]. The Xiaomi Mi Band is not a device that is frequently used in scientific research, unlike other devices, such as those manufactured by Fitbit [4,7,9]. However, recent studies have used the Xiaomi Mi Band as an objective tool to record physical activity and sleep in older populations [43-45] and work environments [46,47]. Moreover, previous research compared the sleep data of Xiaomi devices, specifically the Xiaomi Mi Bands 2 and 3, with other devices, concluding that the Xiaomi devices do not correctly identify the TST and WASO periods [9,30]. Nevertheless, researchers have not comprehensively assessed the Xiaomi Mi Band against PSG and specifically analyzed how it records sleep stages in people with SDis as well as those without SDis. Therefore, this study is the first to address validation of the Xiaomi Mi Band 5 against PSG.

### Objectives

This study aimed to compare the performance of the Xiaomi Mi Band 5 in measuring the sleep-wake stages compared with PSG performed at a hospital sleep unit. The secondary objectives were (1) to determine the agreement between sleep measures from PSG and the Xiaomi Mi Band 5; (2) to assess the accuracy, specificity, and sensitivity for classifying sleep and wake stages by the Xiaomi Mi Band 5 compared with PSG; and (3) to determine the performance level of the Xiaomi Mi Band 5 for detecting sleep stages (wake, *light sleep*, *deep sleep*, and REM sleep) compared with PSG.

## Methods

### Participants

This study was carried out from August 4, 2020, to December 10, 2021. Participants were recruited from the sleep unit of a hospital in A Coruña, Spain. These people were attending the sleep unit to participate in a PSG study, intended to detect possible sleep alterations, independent of this project. All participants had access to a study information sheet and provided written informed consent for their participation. The protocol study was registered with the ClinicalTrials.gov Protocol Registration and Results System (NCT04568408) and published in an international journal, where the design and recruitment process of the study are detailed [48]. The research group maintained the anonymization of all data recorded and obtained from each participant, following and respecting the European (UE 2018/1725) and Spanish (BOE-A-2018-16673) laws on personal data protection at all times.

A total of 58 people participated in the Xiaomi Mi Band 5 validation project. However, data from 13 (22%) of the 58 participants were not used in the sleep analysis owing to different factors, such as Xiaomi device malfunction (10/13, 77%), not meeting the inclusion criteria (2/13, 15%), and not performing a PSG (1/13, 8%). Therefore, the final sample comprised 45 people (n=23, 51%, men and n=22, 49%, women; aged 23-81 [mean 53.24 SD 15.44] years; BMI mean 27.86, SD 4.44 kg/m^2^). Of these 45 participants, 25 (56%) were diagnosed with SDis after they had undergone PSG. The sleep diagnoses were OSA syndrome (18/25, 72%), insomnia (4/25, 16%), narcoleptic syndrome (1/25, 4%), a combination of hypersomnia and narcoleptic syndrome (1/25, 4%), and a combination of sleep apnea syndrome and hypoventilation syndrome (1/25, 4%). For this reason, the sleep measures were also analyzed in 2 groups according to whether there were SDis. The demographic characteristics of the No SDis (20/45, 44%) and SDis (25/45, 56%) groups are shown in Table 1.

**Table 1:** Sample characteristics (n=45).

Characteristics			No SDis^a^ group (n=20)		SDis group (n=25)
**Sex, n (%)**					
	Female	12 (60)		10 (40)	
	Male	8 (40)		15 (60)	
Age (years), mean (SD)			49.7 (14.22)		56.08 (16.06)
BMI (kg/m^2^), mean (SD)			26.55 (3.67)		28.91 (4.79)
PSQI^b^, mean (SD)			10.30 (4.66)		9.8 (4.07)

^a^SDis: sleep disorders.

^b^PSQI: Pittsburgh Sleep Quality Index.

### Procedure

All participants slept in the sleep unit for 1 night. On the day of the recording, participants did not drink liquids for 3 hours before PSG, and they attended the sleep unit a few hours earlier to become acquainted with the bedroom. The technical team members of the sleep unit were in charge of supervising, preparing the participants for PSG, and meeting their possible demands. Regarding PSG, the technicians placed the sensors with electrode gel on the participants and connected them to start the test. Participants wore the Xiaomi Mi Band 5 during the recording test, and its location on the wrist was noted by the technicians. During the registration, the technical team members supervised the PSG and the Xiaomi device worn by the participants, making notes and marking alterations that emerged throughout the night for subsequent analysis. The lights-off and lights-on times, temperature, and sound were controlled in the room by the technicians. PSG and Xiaomi data were collected and synchronized simultaneously. The data coincided with the lights-off and lights-on times, providing a record of approximately 8 hours of time in bed (TIB).

### PSG Assessment

PSG was performed using the NicoletOne v44 Sleep Diagnostic System (Natus Medical Incorporated). This device uses several recordings that included an electroencephalogram (EEG; 6 leads: FP1/FP2, F3/F4, C3/C4, and O1/O2 referenced by the contralateral mastoid), a submental (P3 and P4) and bilateral anterior tibial (2 electrodes on each leg to assess leg movements) electromyogram (EMG), a bilateral electro-oculogram (EOG), and an electrocardiogram (ECG) [16]. EEG, EMG, EOG, and ECG signals were sampled at 256 Hz. The EEG and EOG signals were filtered at 0.3 to 35 Hz, the EMG signal was filtered at 10 to 100 Hz, and the ECG signal was filtered at 0.3 to 70 Hz. At the same time, other biomedical parameters such as respiratory movements and efforts (thoracic and abdominal bands), nasal and oral airflow (nasal cannula), arterial oxygen saturation (pulse oximeter), cardiac activity, and body movement band were recorded to provide relevant information for a potential diagnosis of SDis [16]. PSG parameters were interpreted by the specialized physician of the sleep unit to obtain the cycles and stages of sleep (wake time, N1 sleep, N2 sleep, N3 sleep, and REM sleep) and were scored in 30-second epochs according to the standards of the AASM [49].

### Xiaomi Mi Band 5

The Xiaomi Mi Band 5 includes a 3-axis accelerometer, a 3-axis gyroscope, an HR sensor, and a photoplethysmography sensor to measure some biomedical parameters. This device contains updated software that continuously records daily activity (eg, steps, distance, activity time, and calories), sleep (eg, *light sleep*, *deep sleep*, REM sleep, wake, TST, start and end of the sleep period, and naps), and HR [50]. The device also calculates and classifies the stress level (classified using the terms *relaxed*, *mild*, *moderate*, and *high*) through HR data. In addition, the wristband requires a series of personal data such as age, sex, weight, height, handedness, and wristband location. The Xiaomi Mi Band connects to its app, the Zepp Life app, via Bluetooth, where the recorded data are transferred and displayed [50]. The Zepp Life app allows the export of activity data (broken down into total activity, minute-by-minute activity, and activity stage), sleep data (start and end sleep periods, WASO, *light sleep*, *deep sleep*, and REM sleep), HR data (broken down into total HR; minimum, maximum, and mean HR; and minute-by-minute HR), sports data, and body data in CSV files [50]. In this study, some modes of the Xiaomi device were activated, such as automatic HR, sleep assistant, and night mode, to obtain accurate data and to not disturb the participant. Moreover, there were no problems with the charging of the wristband battery or with the battery itself.

### Processing the PSG and Xiaomi Mi Band 5 Data

The Xiaomi Mi Band 5 and PSG source data were originally available in different formats. The Xiaomi Mi Band 5 data were collected from the Zepp Life app manually and exported to an Excel sheet (Microsoft Corp) because it did not allow downloading of the raw data from the wristband. By contrast, PSG data consisted of CSV text files that contained the manually scored sleep stages and the corresponding reports with clinical sleep diagnostic parameters, available in Word format (Microsoft Corp), both exported using the NicoletOne software. Hence, to enable performance analysis, data were converted into a common format using the European Data Format + (EDF+) [51]. For this purpose, a Python script (version 3.10.5; Python Software Foundation) was developed with help of the *PyEDFlib* library [52].

To carry out this process, *start time*, *lights off*, *lights on*, and *end of the test* markers were set according to the expert annotations available in the corresponding PSG reports. Sleep stages were coded using EDF+ standard texts following the AASM guidelines [49]. For the Xiaomi Mi Band 5 data, it was necessary to adjust the resolution to standard 30-second epochs because the device originally reported on the basis of 1-minute epochs only. This was achieved by splitting each 1-minute epoch into 2 corresponding epochs of 30 seconds each sharing the same sleep stage. Likewise, the Xiaomi hypnogram information does not consider N1 and N2 stages separately. For this reason, epoch-by-epoch (EBE) performance analysis was carried out using a 4-way classification (wake, *light sleep*, *deep sleep* or N3, and REM sleep) by merging the corresponding N1 and N2 labels from the PSG in the corresponding *light sleep* category. Performance analysis was carried out using information within the TIB periods. It should be noted that the Xiaomi Mi Band 5 only starts reporting after the first sleep period is identified by the device; therefore, it is assumed that the periods before the first sleep period and after the last sleep period detected by the device within the TIB period are scored as wakefulness.

After this process, and using the corresponding EDF+ annotation files, different standard sleep parameters were calculated and compared [53]. More specifically, TIB (calculated in min), total sleep period duration (TSPD; a measure containing the duration of both sleep and wake cycles; min), TST (min), WASO (min), awakenings (number), SOL (min), SE (percentage), *light sleep* (min), *deep sleep* (min), REM sleep (min), and awake (min) were analyzed for both the Xiaomi Mi Band 5 and PSG in this study. Normative values for some of these parameters have been proposed based on the consensus of a panel of experts regarding objective assessment of sleep quality [41].

### Statistical Analysis

Statistical analysis was performed using R software (version 4.1.2; R Foundation for Statistical Computing). The analysis was carried out with the total sample (n=45). Likewise, data were analyzed with the participants grouped depending on the existence of SDis to determine the differences between PSG and the Xiaomi Mi Band 5.

Summary measures of PSG and the Xiaomi Mi Band 5 equivalents were compared using the paired 2-tailed *t* test [54] or the Mann-Whitney Wilcoxon test [54]. The choice of the test was based on whether the data were normally distributed, determined by using the Shapiro-Wilk normality test. Normally distributed data were analyzed using the parametric 2-tailed *t* test, whereas nonnormally distributed data were analyzed using the Mann-Whitney Wilcoxon test. Moreover, the effect size was measured using Cohen *d*, classified as 0.2 (small effect), 0.5 (moderate effect), and 0.8 (large effect) [54].

Furthermore, the Bland-Altman method was used to determine the agreement between PSG and the Xiaomi Mi Band 5 for each sleep parameter. The mean difference (or bias) between the methods, the SD, the 95% CI, and the Bland-Altman 95% limits of agreement (mean observed difference ± 1.96 × SD of observed differences) were calculated. A positive bias indicates that the Xiaomi Mi Band 5 tends to underestimate a variable when compared with the gold standard (PSG). A negative bias indicates that a sleep variable is overestimated [55].

EBE analysis was performed according to the 2 levels of compliance as provided in the ANSI and CTA performance evaluation guidelines [33]. The first level analyzed the performance of the devices in a 2-way classification for detecting sleep-wake stages using a confusion matrix. For this purpose, accuracy (proportion of correctly classified sleep and wake epochs), sensitivity (proportion of epoch segments identified as sleep by the Xiaomi Mi Band 5 of those classified as sleep by the PSG), specificity (proportion of epoch segments identified as wake by the Xiaomi Mi Band 5 of those classified as wake by PSG), and Cohen κ values (agreement corrected by chance between the Xiaomi Mi Band 5 and PSG) were analyzed [33]. The standard label definitions used to classify Cohen κ values were used: 0 to 0.2 (slight), 0.21 to 0.40 (fair), 0.41 to 0.60 (moderate), 0.61 to 0.80 (substantial), and >0.80 (almost perfect) [56]. The second level analyzed how the device detected sleep stages using a 4-way classification (wake, REM sleep, *light sleep*, and *deep sleep*). For this purpose, accuracy and Cohen κ values were analyzed [33].

### Ethics Approval

The study was approved by the A Coruña-Ferrol research ethics committee (2020/318).

## Results

### Comparison of PSG and Xiaomi Mi Band 5 Sleep Measures

The sleep parameters obtained from PSG and the Xiaomi Mi Band 5 were compared using the 2-tailed *t* test or the Mann-Whitney Wilcoxon test. Table 2 provides the sleep outcomes for PSG and the Xiaomi Mi Band 5 in the total sample. Overall, there were no significant differences between the methods in the *initial sleep onset* (*P*=.27), TSPD (*P*=.78), and SOL (*P*=.29) measures. However, the Xiaomi Mi Band 5 significantly overestimated PSG TST (*z*=−2.73; *P*=.009), the percentage of PSG SE (*z*=−2.59; *P*=.03), PSG *light sleep* (t_44_=−2.49; *P*=.005), and PSG *deep sleep* (t_44_=−2.58; *P*=.01). In addition, it underestimated PSG WASO (*z*=2.96; *P*=.005), PSG awakenings (*z*=7.72; *P*=.001), PSG REM sleep (t_44_=3.59; *P*=.001), and PSG awake (*z*=2.61; *P*=.01).

Table 3 shows the results of sleep measures for PSG and the Xiaomi Mi Band 5 in the No SDis and SDis groups. There were no significant differences between PSG and the Xiaomi Mi Band 5 for the *initial sleep onset* (No SDis: *P*=.37; SDis: *P*=.52) and TSPD (No SDis: *P*=.47; SDis: *P*=.57) variables in either group. However, the Xiaomi Mi Band 5 differed significantly from PSG in the rest of the sleep measures, depending on whether the participants presented or did not present SDis. In the No SDis group, the Xiaomi Mi Band 5 overestimated PSG SOL (z=−1.83; *P*=.046) and PSG *light sleep* (t_19_=−3.23; *P*=.004), and it underestimated PSG awakenings (z=5.50; *P*<.001) and PSG REM sleep (t_19_=2.66; *P*=.02). By contrast, in the SDis group, it overestimated PSG TST (z=−2.70; *P*=.007), the percentage of PSG SE (z=−2.19; *P*=.03), and PSG *light sleep* (t_19_=−3.97; *P*<.001), and it underestimated PSG WASO (z=2.35; *P*=.02), PSG awakenings (z=5.35; *P*<.001), PSG REM sleep (t_19_=2.37; *P*=.03), and PSG awake time (z=3.13; *P*=.005).

**Table 2:** Comparison of polysomnography (PSG) and Xiaomi Mi Band 5 sleep measures in the total sample.

	PSG, mean (SD; 95% CI)	Xiaomi Mi Band 5, mean (SD; 95% CI)	*t* test (*df*)	*z* score^a^	*P* value	Cohen *d*
Lights on (hh:mm)	07:01 (00:17; 06:56-07:10)	—^b^	—	—	—	—
Lights off (hh:mm)	23:36 (00:40; 22:29-01:03)	—	—	—	—	—
Initial sleep onset (hh:mm)	00:29 (01:31; 22:47-01:33)	00:11 (01:05; 23:55-00:31)	1.12 (44)	—	.27	0.208^c^
TIB^d^ (min)	443.58 (44.98; 430.07 to457.1)	—	—	—	—	—
TSPD^e^, (min)	408.05 (57.13; 390.88-425.23)	406.16 (51.27; 390.75-421.56)	0.28 (44)	—	.78	0.250^c^
TST^f^ (min)	344.62 (79.58; 320.71-368.53)	374.17 (72.42; 352.42-395.93)	—	−2.73	.009	−0.407^c^
WASO^g^ (min)	63.42 (57.42; 46.17 to80.68)	31.99 (51.64; 16.47-47.50)	—	2.96	.005	0.442^c^
Awakenings (>5 min; number per night)	3.64 (2.27; 4.33-2.96)	0.69 (0.94; 0.97-0.40)	—	7.72	.001	1.15^h^
SOL^i^ (min)	31.64 (33.97; 21.43-41.84)	40.26 (42.59; 27.47-53.06)	—	−1.07	.29	−0.206^c^
SE^j^ (%)	78.32 (16.56; 73.35-83.30)	84.14 (15.70; 79.42-88.86)	—	−2.59	.03	−0.329^c^
Time in N1 stage of non-REM^k^ sleep (min)	12.65 (9.21; 9.9-15.42)	—	—	—	—	—
Time in N2 stage of non-REM sleep (min)	202.14 (57.47; 184.87-219.40)	—	—	—	—	—
Time in N1+N2 sleep (light sleep; min)	214.79 (55.12; 198.23-231.35)	244.62 (56.59; 227.61-261.62)	−2.49 (44)	—	.005	−0.439^c^
Time in N3 sleep (deep sleep; min)	60.72 (29.41; 51.88-69.56)	75.37 (31.73; 65.83-84.90)	−2.58 (44)	—	.01	−0.385^c^
Time in REM sleep (min)	69.11 (28.08; 60.67-77.54)	49.61 (30.7; 40.01-59.22)	3.59 (44)	—	.001	0.536^l^
Awake (min)	94.86 (71.88; 116.46-73.27)	66.51 (66.22; 86.40-46.61)	—	2.61	.01	0.390^c^

^a^Mann-Whitney Wilcoxon test.

^b^Not available.

^c^Small effect.

^d^TIB: time in bed.

^e^TSPD: total sleep period duration.

^f^TST: total sleep time.

^g^WASO: wake after sleep onset.

^h^Large effect.

^i^SOL: sleep onset latency.

^j^SE: sleep efficiency.

^k^REM: rapid eye movement.

^l^Moderate effect.

**Table 3:** Comparison of polysomnography (PSG) and Xiaomi Mi Band 5 sleep measures in the no sleep disorders (No SDis) and sleep disorders (SDis) groups.

		PSG, mean (SD; 95% CI)	Xiaomi Mi Band 5, mean (SD; 95% CI)	*t* test (*df*)	*z* score^a^	*P* value	Cohen *d*
**Lights on (hh:mm)**							
	No SDis group	07:02 (00:11; 06:57-07:07)	—^b^	—	—	—	—
	SDis group	07:01 (00:45; 06:52-07:10)	—	—	—	—	—
**Lights off (hh:mm)**							
	No SDis group	23:32 (00:48; 23:09-23:54)	—	—	—	—	—
	SDis group	23:42 (00:32; 23:28-23:55)	—	—	—	—	—
**Initial sleep onset (hh:mm)**							
	No SDis group	00:37 (02:00; 23:41-01:33)	00:10 (00:56; 23:43-00:37)	0.91 (19)	—	.37	0.204^c^
	SDis group	00:22 (01:02; 23:56-00:48)	00:12 (01:13; 23:42-00:43)	0.64 (24)	—	.52	0.230^c^
**TIB^d^ (min)**							
	No SDis group	446.66 (50.37; 423.09-470.24)	—	—	—	—	—
	SDis group	441.12 (41.06; 424.16-458.07)	—	—	—	—	—
**TSPD^e^ (min)**							
	No SDis group	442.80 (42.35; 402.97-442.62)	410.56 (50.66; 386.84-434.27)	2.12 (19)	—	.47	0.475^c^
	SDis group	396.26 (65.11; 369.38-423.13)	402.64 (52.51; 380.96-424.32)	−0.58 (24)	—	.57	−0.508^f^
**TST^g^ (min)**							
	No SDis group	363.55 (68.20; 331.63-395.47)	378.26 (70.98; 345.04-411.49)	−0.83 (19)	—	.41	−0.208^c^
	SDis group	329.48 (85.97; 293.99-364.97)	370.90 (74.84; 340.00-401.79)	—	−2.70	.007	−0.625^f^
**WASO^h^ (min)**							
	No SDis group	59.25 (56.44; 32.83-85.66)	32.30 (54.31; 6.87-57.71)	—	1.98	.12	0.361^c^
	SDis group	66.77 (59.14; 42.36-91.18)	31.75 (50.53; 10.89-52.61)	—	2.35	.02	0.504^f^
**Awakenings (>5 min; number per night)**							
	No SDis group	3.90 (2.38; 5.01-2.78)	0.65 (0.98; 1.11-0.18)	—	5.50	<.001	1.20^i^
	SDis group	3.44 (2.22; 4.35-2.52)	0.72 (0.93; 1.10-0.33)	—	5.35	<.001	1.07^i^
**SOL^j^ (min)**							
	No SDis group	26.47 (15.19; 19.36-33.58)	38.75 (32.46; 23.56-53.95)	—	−1.83	.05	−0.478^c^
	SDis group	35.78 (43.50; 17.82-53.73)	41.48 (49.88; 20.89-62.07)	—	−0.25	.80	−0.474^c^
**SE^k^ (%)**							
	No SDis group	81.02 (13.67; 74.63-87.42)	84.61 (15.24; 77.47-91.74)	—	−1.46	.37	−0.206^c^
	SDis group	76.17 (18.56; 68.51-83.83)	83.77 (16.36; 77.02-90.53)	—	−2.19	.03	−0.421^c^
**Time in N1 stage of non-REM^l^ sleep (min)**							
	No SDis group	13.85 (10.05; 9.14-18.55)	—	—	—	—	—
	SDis group	11.70 (8.56; 8.16-15.23)	—	—	—	—	—
**Time in N2 stage of non-REM sleep (min)**							
	No SDis group	198.74 (52.72; 174.06-223.42)	—	—	—	—	—
	SDis group	204.86 (61.95; 179.28-230.43)	—	—	—	—	—
**Time in N1+N2 sleep (light sleep; min)**							
	No SDis group	212.59 (48.09; 190.08-235.10)	254.63 (52.78; 229.93-279.33)	−3.23 (19)	—	.004	−0.724^f^
	SDis group	216.56 (61.09; 191.34-241.77)	236.59 (59.29; 212.12-261.07)	−1.34 (24)	—	.19	−0.268^c^
**Time in N3 sleep (deep sleep; min)**							
	No SDis group	77.05 (20.68; 67.37-86.73)	73.88 (30.54; 59.59-88.17)	0.42 (19)	—	.67	−0.345^c^
	SDis group	47.66 (29.11; 35.64-59.67)	76.55 (33.22; 62.84-90.27)	−3.97 (24)	—	<.001	−0.796^f^
**Time in REM sleep (min)**							
	No SDis group	73.90 (23.56; 62.88-84.94)	49.85 (27.20; 37.12-62.58)	2.66 (19)	—	.02	0.597^f^
	SDis group	65.26 (31.17; 52.39-78.13)	49.43 (35.89; 34.62-64.24)	2.37 (24)	—	.03	0.475^c^
**Awake (min)**							
	No SDis group	85.72 (62.81; 115.12-56.33)	71.01 (73.04; 105.21-36.83)	—	0.83	.42	0.218^c^
	SDis group	102.18 (78.90; 134.75-69.61)	60.77 (62.39; 86.53-35.01)	—	3.13	.005	0.625^f^

^a^Mann-Whitney Wilcoxon test.

^b^Not available.

^c^Small effect.

^d^TIB: time in bed.

^e^TSPD: total sleep period duration.

^f^Moderate effect.

^g^TST: total sleep time.

^h^WASO: wake after sleep onset.

^i^Large effect.

^j^SOL: sleep onset latency.

^k^SE: sleep efficiency.

^l^REM: rapid eye movement.

### Bland-Altman Plots

**Figure 1:**
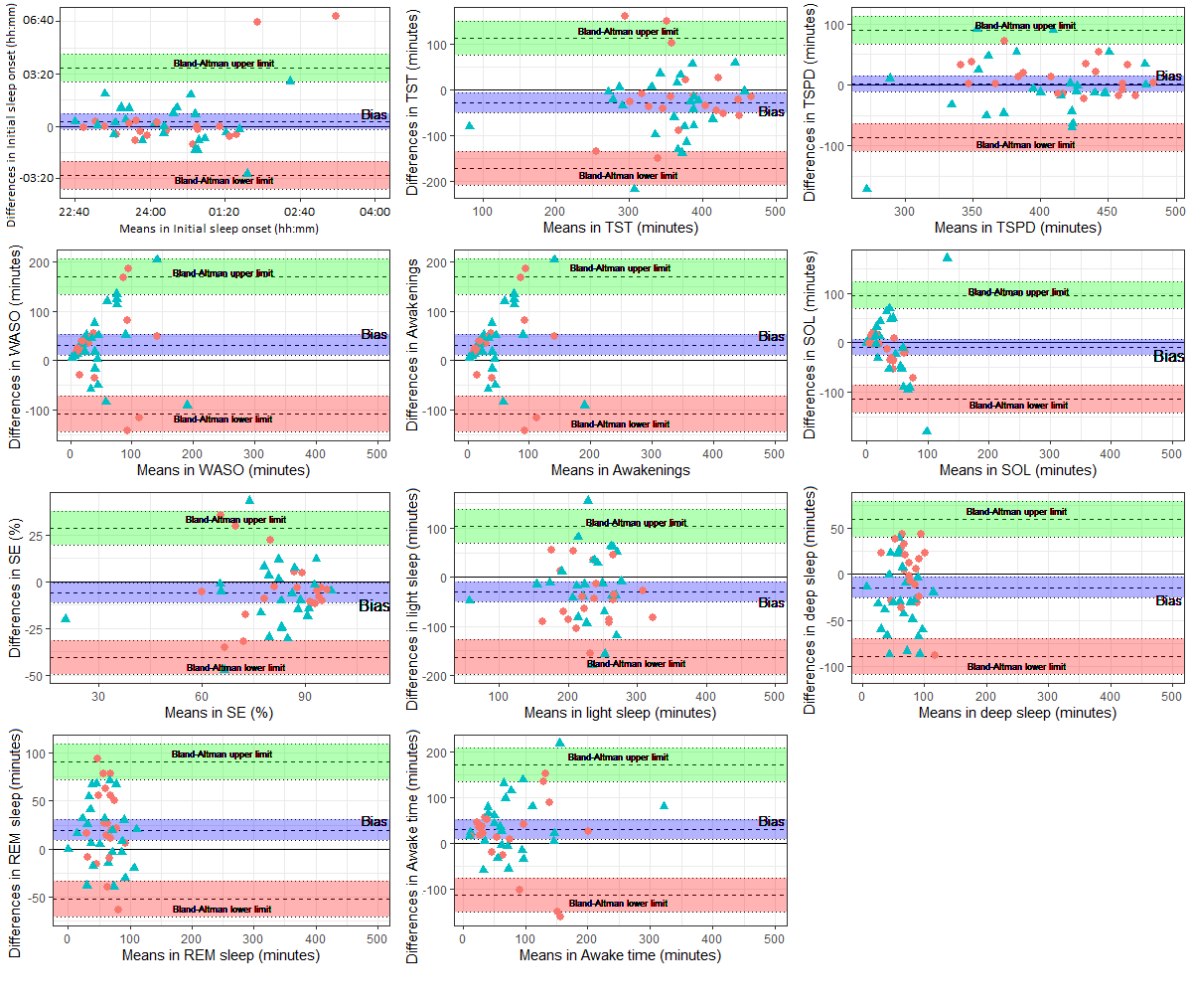
Bland-Altman plots for initial sleep onset, total sleep time (TST), total sleep period duration (TSPD), wake after sleep onset (WASO), awakenings, sleep onset latency (SOL), sleep efficiency (SE), light sleep, deep sleep, rapid eye movement (REM) sleep, and awake time. The PSG-Xiaomi Mi Band 5 differences for sleep parameters (y-axis) are plotted as a function of the PSG-Xiaomi Mi Band 5 means (x-axis) for sleep parameters. Circles represent participants without sleep disorders (No SDis group; n=20), and triangles represent participants with sleep disorders (SDis group; n=25). Zero lines are marked and represent perfect agreement. The dotted lines represent the biases and Bland-Altman 95% limits of agreement (mean observed difference ± 1.96 × SD of observed differences). PSG: polysomnography.

Table 4 shows Bland-Altman biases, SDs, 95% CIs, and the upper and lower Bland-Altman 95% limits of agreement. Figure 1 presents Bland-Altman plots for the main sleep measures. In the total sample, the Xiaomi Mi Band 5 significantly overestimated PSG TST by 29.54 minutes, PSG SE by 5.82%, PSG *light sleep* by 29.81 minutes, and PSG *deep sleep* by 14.64 minutes. By contrast, it underestimated PSG WASO by 31.44 minutes, PSG awakenings by 2.95 epochs, PSG REM sleep by 19.49 minutes, and PSG awake time by 28.36 minutes.

There were also differences between the sleep groups. The Xiaomi Mi Band 5 significantly overestimated PSG *light sleep* by 42.02 minutes and PSG SOL by 12.28 minutes in the No SDis group. In addition, it underestimated PSG awakenings by 3.25 epochs and PSG REM sleep by 24.05 minutes in this group. However, in the SDis group, the Xiaomi Mi Band 5 overestimated PSG TST by 41.41 minutes, PSG SE by 7.6%, and PSG *deep sleep* by 28.89 minutes. In addition, it underestimated PSG WASO by 35.03 minutes, PSG awakenings by 2.72 epochs, PSG REM sleep time by 15.83 minutes, and PSG awake time by 41.41 minutes.

On average, the Bland-Altman agreement limits were exceeded every 4 and 2 participants for the total sample, especially in TST, WASO, awakenings, and SE measures. The participants with sleep problems were the ones who mainly exceeded these aggregation limits.

**Table 4:** Bland-Altman parameters for the comparison between polysomnography and the Xiaomi Mi Band 5 in the total sample as well as the no sleep disorders (No SDis) and sleep disorders (SDis) groups.

		Bias (SD; 95% CI)	Agreement limits	Number of participants exceeding the agreement limits, n (%)
**Initial sleep onset (hh:mm)**				
	Total sample	00:17 (01:44; 00:49 to 00:13)	−03:06 to 03:40	2 (4)^a^
	No SDis group	00:27 (02:13; 01:29 to 00:35)	−01:36 to 02:30	2 (10)^b^
	SDis group	00:09 (01:17; 00:41 to 00:21)	−02:22 to 02:40	0 (0)^c^
**TST^d^ (min)**				
	Total sample	−29.54 (72.54; −7.75 to 51.33)	−171.72 to 112.63	3 (7)^a^
	No SDis group	−14.71 (78.94; −51.66 to 22.23)	−169.44 to 140.01	1 (5)^b^
	SDis group	−41.41 (66.20; −68.73 to 14.08)	−171.18 to 88.36	2 (8)^c^
**TSPD^e^ (min)**				
	Total sample	1.89 (44.97; −11.61 to 15.40)	−86.25 to 90.04	1 (2)^a^
	No SDis group	12.24 (25.75; 0.19 to 24.29)	−38.24 to 62.73	0 (0)^b^
	SDis group	−6.38 (54.97; −29.07 to 16.31)	−114.14 to 101.37	1 (4)^c^
**WASO^f^ (min)**				
	Total sample	31.44 (71.13; 10.07 to 52.81)	−107.97 to 170.86	4 (9)^a^
	No SDis group	26.96 (61.92; −8.00 to 61.93)	−119.47 to 173.40	1 (5)^b^
	SDis group	35.03 (69.48; 6.35 to 63.71)	−101.15 to 171.21	3 (12)^c^
**Awakenings (>5 min; number per night)**				
	Total sample	2.95 (2.57; 3.73 to 2.18)	−2.07 to 3.73	4 (9)^a^
	No SDis group	3.25 (2.63; 4.48 to 2.01)	−1.91 to 8.41	3 (15)^b^
	SDis group	2.72 (2.54; 3.77 to 1.67)	−2.26 to 7.70	1 (4)^c^
**SOL^g^ (min)**				
	Total sample	−8.62 (53.76; −24.77 to 7.53)	−114.00 to 96.75	2 (4)^a^
	No SDis group	−12.28 (25.69; −24.31 to 0.26)	−62.65 to 38.08	0 (0)^b^
	SDis group	−5.7 (68.97; −34.17 to 22.77)	−140.88 to 129.48	2 (8)^c^
**SE^h^ (%)**				
	Total sample	−5.82 (17.67; −11.13 to 0.51)	−40.46 to 28.82	4 (9)^a^
	No SDis group	−3.85 (17.37; −11.71 to 4.54)	−37.63 to 30.46	2 (10)^b^
	SDis group	−7.60 (18.06; −15.06 to 0.14)	−43.00 to 27.80	2 (8)^c^
**Time in N1+N2 sleep (light sleep; min)**				
	Total sample	−29.81 (67.98; −50.24 to 9.39)	−163.07 to 103.44	2 (4)^a^
	No SDis group	−42.04 (58.04; −69.20 to 14.87)	−155.81 to 71.72	0 (0)^b^
	SDis group	−20.03 (74.72; −50.88 to 10.81)	−166.49 to 126.42	2 (8)^c^
**Time in N3 sleep (deep sleep; min)**				
	Total sample	−14.64 (59.97; −26.08 to 3.20)	−89.27 to 59.98	0 (0)^a^
	No SDis group	3.17 (33.00; −12.28 to 18.62)	−61.53 to 67.86	0 (0)^b^
	SDis group	−28.89 (36.32; −43.88 to 13.90)	−100.08 to 36.32	0 (0)^c^
**Time in REM^i^ sleep (min)**				
	Total sample	19.49 (36.40; 8.55 to 30.42)	−51.85 to 90.84	2 (4)^a^
	No SDis group	24.05 (40.30; 5.20 to 42.92)	−54.93 to 103.05	0 (0)^b^
	SDis group	15.83 (33.34; 2.07 to 29.60)	−49.52 to 81.20	2 (8)^c^
**Awake (min)**				
	Total sample	28.36 (72.69; 50.20 to 6.52)	−114.11 to 170.84	3 (7)^a^
	No SDis group	14.71 (78.93; 51.65 to 22.23)	−140.00 to 169.43	2 (10)^b^
	SDis group	41.41 (66.21; 14.08 to 68.74)	−88.36 to 171.18	1 (4)^c^

^a^n=45.

^b^n=20.

^c^n=25.

^d^TST: total sleep time.

^e^TSPD: total sleep period duration.

^f^WASO: wake after sleep onset.

^g^SOL: sleep onset latency.

^h^SE: sleep efficiency.

^i^REM: rapid eye movement.

### EBE Analysis

Tables 5-7 show the confusion matrices resulting from the 2-way (wake vs sleep) classification of 30-second epochs between PSG and the Xiaomi Mi Band 5. Results are shown separately for the total sample as well as for the No SDis and SDis groups.

**Table 5:** Confusion matrix for the 2-way (wake vs sleep) epoch-by-epoch classification for the total sample.

		Xiaomi Mi Band 5	
		Wake	Sleep
**PSG^a^**			
	Wake	3019	5525
	Sleep	3238	27,786

^a^PSG: polysomnography.

**Table 6:** Confusion matrix for the 2-way (wake vs sleep) epoch-by-epoch classification for the no sleep disorders group.

		Xiaomi Mi Band 5	
		Wake	Sleep
**PSG^a^**			
	Wake	1305	2117
	Sleep	1528	13,018

^a^PSG: polysomnography.

**Table 7:** Confusion matrix for 2-way (wake vs sleep) epoch-by-epoch classification for the sleep disorders group.

		Xiaomi Mi Band 5	
		Wake	Sleep
**PSG^a^**			
	Wake	1714	3408
	Sleep	1710	14,786

^a^PSG: polysomnography.

According to the results, the Xiaomi Mi Band 5 correctly classified both sleep and wake epochs in 30,805 (77.85%) of the 39,568 available epochs. It correctly detected 27,786 (89.56%) of the 31,024 sleep epochs, thus resulting in 0.90 sensitivity for the sleep class, and it was able to identify 3019 (35.33%) of the 8544 wake stages, leading to a corresponding 0.35 specificity for the wake class. Because of the binary classification, we derive immediately the respective sensitivity and specificity values for the wake class as 0.35 and 0.90. On the basis of the Cohen κ value, the level of concordance between PSG and the Xiaomi Mi Band 5 was 0.22.

These results were similar in the No SDis and SDis groups. The Xiaomi Mi Band 5 had an accuracy of 0.80 and 0.76 in the No SDis group and the SDis group, respectively. The sensitivity for the sleep class was 0.89 for both groups, and the corresponding sensitivity was 0.38 for the No SDis group and 0.33 for the SDis group. The Cohen κ values for the Xiaomi Mi Band 5 were 0.27 in the No SDis group and 0.26 in the SDis group.

Tables 8-10 show the corresponding confusion matrices for the 4-way sleep stage classification between PSG and the Xiaomi Mi Band 5. In general, the accuracy level was 0.44. More specifically, the agreement with PSG was 0.48 for the detection of wakefulness, 0.51 for *light sleep*, 0.33 for *deep sleep*, and 0.26 for REM sleep. The No SDis and SDis groups had outcomes similar to those of the total sample. Moreover, the Xiaomi Mi Band 5 misidentified PSG epochs 40% to 70% of the time. It should be noted that the Xiaomi device misclassified 3957 (46.31%) of the 8544 wake epochs and 4044 (65.01%) of the 6221 REM sleep stages as *light sleep* in the total sample. In addition, it misclassified 1945 (65.38%) of the 2975 and 2099 (64.31%) of the 3264 REM sleep epochs in the No SDis group and the SDis group, respectively, as *light sleep*.

Table 11 shows that the Cohen κ coefficients for the 4-way epoch classification ranged between 0.11 and 0.15 for agreement between PSG and the Xiaomi Mi Band 5 among the 3 groups, indicating that most of the accuracy agreement was due to chance.

**Table 8:** Confusion matrix for 4-way (wake, light sleep, deep sleep, and rapid eye movement [REM] sleep) epoch-by-epoch classification for the total sample.

			Xiaomi Mi Band 5						
			Wake		Light sleep		Deep sleep		REM
**PSG^a^**									
	Wake	3019		3957		1020		548	
	Light sleep	2236		11,366		3468		2274	
	Deep sleep	537		2653		1775		494	
	REM	465		4044		519		1193	

^a^PSG: polysomnography.

**Table 9:** Confusion matrix for 4-way (wake, light sleep, deep sleep, and rapid eye movement [REM] sleep) epoch-by-epoch classification for the no sleep disorders group.

			Xiaomi Mi Band 5						
			Wake		Light sleep		Deep sleep		REM
**PSG^a^**									
	Wake	1305		1630		272		215	
	Light sleep	872		5142		1438		1055	
	Deep sleep	422		1469		982		209	
	REM	234		1945		263		515	

^a^PSG: polysomnography.

**Table 10:** Confusion matrix for 4-way (wake, light sleep, deep sleep, and rapid eye movement [REM] sleep) epoch-by-epoch classification for the sleep disorders group.

			Xiaomi Mi Band 5						
			Wake		Light sleep		Deep sleep		REM
**PSG^a^**									
	Wake	1714		2327		748		333	
	Light sleep	1364		6224		2030		1219	
	Deep sleep	115		1184		793		285	
	REM	231		2099		256		678	

^a^PSG: polysomnography.

**Table 11:** Overall accuracy and Cohen κ statistics for 2-way and 4-way epoch-by-epoch classifications for the total sample as well as the no sleep disorders (No SDis) and sleep disorders (SDis) groups.

	2-Way epoch-by-epoch classification			4-Way epoch-by-epoch classification	
	Accuracy, mean (SD)	Cohen κ^a^, mean (SD)	Accuracy, mean (SD)		Cohen κ, mean (SD)
Total sample	0.78 (0.13)	0.22 (0.23)	0.44 (0.10)		0.12 (0.13)
No SDis	0.80 (0.13)	0.27 (0.21)	0.45 (0.10)		0.15 (0.12)
SDis	0.76 (0.12)	0.26 (0.25)	0.43 (0.10)		0.11 (0.14)

^a^Cohen κ: 0 to 0.2 (slight), 0.21 to 0.40 (fair), 0.41 to 0.60 (moderate), 0.61 to 0.80 (substantial), and >0.80 (almost perfect).

## Discussion

### Principal Findings

The Xiaomi Mi Band 5 is one of the most popular wristbands among consumers around the world [4,5]. Its use has increased because it can continuously record different parameters, including sleep [4,5]. Nevertheless, it was not developed for clinical or scientific purposes in the diagnosis of SDis or sleep monitoring, a factor that could influence its performance [11,28]. Thus, we considered it necessary to determine the quality and accuracy of sleep data obtained from this device by comparing it against PSG performed at a clinical sleep unit. To our knowledge, this is the first study that has validated the ability of the Xiaomi Mi Band 5 to measure sleep parameters in people with SDis as well as those without SDis.

This study investigated the agreement in sleep measures from PSG and the Xiaomi Mi Band 5. Overall, the Xiaomi Mi Band 5 had some limitations in the detection of several sleep measures. There were no significant differences detected among *initial sleep onset*, TSPD, and SOL measures compared with PSG. However, the Xiaomi Mi Band 5 significantly overestimated TST, SE, *light sleep*, and *deep sleep*. It also significantly underestimated WASO, the number of awakenings, REM sleep, and awake time. These results are similar to those of previous studies that validated activity wristbands such as the Fitbit Alta HR [34], Fitbit Charge HR [57], Fitbit Charge 2 [37], and Jawbone UP [38].

The Bland-Altman analysis showed the biases between the Xiaomi Mi Band 5 and PSG in general, which ranged from 1.89 to 31.44 minutes. Unlike other devices, the Xiaomi Mi Band 5 more accurately estimated some summary measures of sleep compared with PSG (Multimedia Appendix 1). PSG TST was overestimated by 25 to 30 minutes more by the Jawbone UP (59.10 min) [38] and the Fitbit Alta HR (53.33 min) [34] than by the Xiaomi Mi Band 5 (29.54 min). Moreover, the Xiaomi Mi Band 5 overestimated PSG *light sleep* by 29.81 minutes, but the Fitbit Charge 2 overestimated it by 34 minutes [37]. Likewise, the Xiaomi Mi Band 5 underestimated PSG WASO by 31.44 minutes and PSG awake time by 28.36 minutes, whereas the Fitbit Alta HR underestimated WASO by 48.37 minutes and awake time by 41.93 minutes [34,36]. By contrast, WASO was underestimated 25.85 minutes [35] less by Fitbit Charge HR (5.6 min) than by the Xiaomi Mi Band 5 (31.44 min).

Furthermore, 2 studies evaluated previous versions of the Xiaomi wristband against other devices [9,30]. However, the authors did not comprehensively compare the sleep parameters of the Xiaomi devices against PSG. Ameen et al [30] used the Xiaomi Mi Band 2 alongside other devices to determine the reliability of sleep data, concluding that the Xiaomi device overestimated TST by 69.64 minutes and SE by 13.25%, and it underestimated WASO by 33.57 minutes. Topalidis et al [9] compared sleep data from the Xiaomi Mi Band 3 and GT3X scientific actigraphy devices, showing low concordance among the devices in the wake periods and TST. Thus, these authors report results that are similar to ours, but TST, SE, and WASO sleep measurement estimates by the Xiaomi Mi Band 5 were more accurate than those in the previous Xiaomi versions [9,30].

The Bland-Altman 95% limits of agreement (Table 4) were generally high, especially for the TST, WASO, the number of awakenings, and SE variables. These limits are similar to those reported in studies that compared the Jawbone UP or the Fitbit Alta HR with PSG [34,38]. However, they differ from those obtained in the Fitbit Charge 2 wristband or the Oura ring validation studies, whose limits of agreement were narrower for all sleep measures [37,39]. In this study, between 9% (4/45) and 4% (2/45) of the participants exceeded the limits of agreement, mainly for the TST, WASO, the number of awakenings, and SE measures. In fact, some of these participants (3/45, 7%) coincide with the disagreement on the limits on these sleep measures. Likewise, several studies have reported a similar number of participants (8%-12% of the participants) who exceeded the aggregation limits [35,38,39].

The Xiaomi Mi Band 5 showed an accuracy of 78% for identifying sleep and wake stages and a sensitivity of 89% for detecting sleep epochs. However, it showed a specificity of only 35%. These findings are similar to those of previous studies, highlighting that, in general, these devices have high accuracy and sensitivity but low specificity [37,38,58]. However, Cohen κ analysis revealed that most of the accuracy was due to chance (Cohen κ=0.22), with 78% (31,024/39,568) of all available epochs in the total sample belonging to the *sleep* class according to PSG. Regarding this aspect, some authors suggest that poor detection of wakefulness time could be due to difficulties the wristbands have in detecting periods of immobility [11,39,59].

Furthermore, this study analyzed the Xiaomi Mi Band 5’s level of performance regarding the identification of sleep stages using a 4-way EBE classification. The device obtained an accuracy of 44% for this task. Specifically, the Xiaomi Mi Band 5 was more accurate in detecting wake (48%) and *light sleep* (51%) than in identifying *deep sleep* (34%) and *REM sleep* (28%), misclassifying these stages as *light sleep* and misclassifying *light sleep* as *REM sleep* on several occasions and, to a lesser extent, as *deep sleep*. Overall, other validated devices seem to have greater accuracy in identifying sleep stages than the Xiaomi Mi Band 5 [11,37,39,60], except for the Fitbit Charge 2, which showed lower accuracy in detecting *deep sleep* (49%) than the Xiaomi Mi Band 5 [37].

Cohen κ corrects the agreement owing to chance between the Xiaomi Mi Band 5 and PSG. Overall, the obtained values hovered between 0.11 and 0.27 for the different 2-way (wake vs sleep) and 4-way stage classifications and patient groups. Thus, the levels of agreement between the Xiaomi Mi Band 5 and PSG were slight to fair. Conversely, other authors reported that the Fitbit Alta HR and the Fitbit Charge 2 devices had Cohen κ coefficients ranging from 0.52 to 0.66, indicating a moderate agreement with PSG [36,37,61].

Experts who have validated other devices have concluded the need to focus on people with sleep problems owing to their increased prevalence during the COVID-19 pandemic [14,62]. The existing literature that validated these devices in people with sleep problems reflects the myriad difficulties that come with measuring sleep parameters [34,36,58]. In this study, the total sample was divided into 2 groups, namely one with SDis and one without SDis. Both groups presented similar results for the *initial sleep onset* and TSPD measures, showing only slight biases with the PSG measures. However, in the rest of the measures, there were relevant differences between the 2 sleep groups. Specifically, the Xiaomi Mi Band 5 showed almost no variation compared with PSG for the TST, SE, *deep sleep*, and awake time variables in the No SDis group. However, in the SDis group, the Xiaomi Mi Band 5 overestimated several parameters compared with PSG, namely TST and *deep sleep* by 28 to 41 minutes and SE by 7.6% in the SDis group. In addition, it underestimated PSG WASO more in the SDis group than in the No SDis group.

The estimations of these sleep measures are consistent with those made by the Jawbone or Fitbit devices in a group of people with SDis [36,38]. However, the Jawbone and Fitbit biases compared with PSG are greater than those obtained in this study [36,38]. Conversely, unlike the No SDis group, the SDis group did not present significant differences with PSG in the variables *light sleep* and SOL. In addition, PSG REM sleep was mainly underestimated in the No SDis group rather than in the SDis group.

Moreover, both groups presented similar results regarding the performance of sleep and wake stage detection, but participants with SDis presented lower values. Overall, Cohen κ coefficients were also lower in the SDis group. Specifically, the Xiaomi Mi Band 5 misidentified more epochs in the SDis group than in the No SDis group. The device misclassified *light sleep* epochs as awake, *deep sleep*, and REM sleep epochs in the SDis group and to a lesser extent in the No SDis group.

Overall, the outcomes obtained from people without SDis were a bit more accurate in some sleep measures and sleep stages. Consistent with the literature, devices such as the Xiaomi Mi Band 5 may be an alternative for health management in people without SDis because the data are more reliable than in people with SDis [8,11,41,61].

However, the outcomes of the SDis group could have been influenced by the inclusion of multiple SDis (rather than a single disorder), with OSA being the most prevalent syndrome. Similar to the results of other studies, the performance of this activity wristband can be lower among people diagnosed with OSA. There are reports that devices such as the Xiaomi Mi Band 5 had worse outcomes in this population than in populations with other conditions [36]. In this study, the biases between the Xiaomi device and PSG differed between participants with OSA and those with other SDis (Multimedia Appendix 2). Our results show better performance in detecting sleep and wake stages, but higher biases were detected for sleep variables in the *other* SDis group.

### Limitations

There are some limitations that could have negatively influenced the main findings of this study. The first limitation is attrition: only 45 of the 58 participants completed the study. Participants did not complete the study owing to difficulties with the use of the wristband and data collection. Hence, the sample was heterogeneous, and the size of the groups (SDis and No SDis) was small. In addition, how the participants were monitored might have influenced the data because this was not done in the usual context in which people sleep. Moreover, participants should be followed up for more days for better assessment of the performance of the Xiaomi Mi Band 5.

This study includes other limitations. Specifically, the Xiaomi Mi Band 5 and other devices only combine movement and HR to classify sleep parameters, whereas PSG includes several sensors; therefore, the accuracy of the data collected by wearable devices could be lower than that of the data collected by PSG. These devices present more limitations in the detection of WASO, *light sleep*, and REM sleep. Moreover, it would be necessary to have access to raw data in 30-second epochs and to the algorithm that Xiaomi uses to classify the data; this additional information would improve data analysis [11]. Although synchronization of the Xiaomi Mi Band 5 and PSG was simultaneous, there may be certain deviations that could affect the results of the study, specifically those obtained in the EBE analysis [11].

### Conclusions

In conclusion, the very popular Xiaomi Mi Band 5 may be an acceptable activity wristband in terms of quality and price. Moreover, its use can promote greater awareness of the importance of sleep and promote good healthy lifestyle habits so that people obtain more quality sleep. Likewise, this device could be considered a tool to monitor sleep and to screen changes in sleep patterns through which health professionals could determine the quality and quantity of people’s sleep. Specifically, it could be a potential tool for use in populations without SDis, especially to identify TST and *deep sleep*. Future research must study the performance of this device in various populations, such as people with OSA, insomnia, narcolepsy, parasomnias, and other health conditions.

## Appendix

Multimedia Appendix 1 Summary of the main findings of the Xiaomi Mi Band 5 and other devices.

Multimedia Appendix 2 Analysis of data according to the type of sleep disorder.

## Abbreviations

AASM: American Academy of Sleep Medicine
ANSI: American National Standards Institute
CTA: Consumer Technology Association
EBE: epoch-by-epoch
ECG: electrocardiogram
EDF+: European Data Format +
EEG: electroencephalogram
EMG: electromyogram
EOG: electro-oculogram
HR: heart rate
OSA: obstructive sleep apnea
PSG: polysomnography
REM: rapid eye movement
SDis: sleep disorders
SE: sleep efficiency
SOL: sleep onset latency
TIB: time in bed
TSPD: total sleep period duration
TST: total sleep time
WASO: wake after sleep onset
