# Supplementary material for: Quality of Sleep Data Validation From the Xiaomi Mi Band 5 Against Polysomnography: Comparison Study
Source: J Med Internet Res. 2023 May 19;25:e42073. doi: 10.2196/42073 (PMC10238963; doi:10.2196/42073)
Supplement: Multimedia Appendix 1 [file jmir_v25i1e42073_app1.docx]

**Quality of Sleep Data Validation from the Xiaomi Mi Band 5 Compared With Polysomnography**

## Appendix 1

**Table S1.** Summary of main findings of Xiaomi and other devices.

| **Comparison sleep measures. Biases (SD)** | | | | | | | | | | | |
| --- | --- | --- | --- | --- | --- | --- | --- | --- | --- | --- | --- |
|  | **Xiaomi** | | | | | **Fitbit** | | | | | **Jabwone UP3** [38] |
|  | **Mi Band 5** | **Mi Band 3**  [9] | | **Mi Band 2** [30] | | **Alta HR**  [34,36] | | **Charge HR**  [35] | **Charge 2** [37] | |  |
| **TST** (minutes) | -29.54 (72.54) | 61 (-) | | 69.64 (67.43) | | 53.33 (-) | | -8 (21) | -9(24) | | 59.1 (-) |
|  |  |  |  |  |  | -59.78 (99.62) | |  |  |  |  |
| **WASO** (minutes) | 31.44 (61.92) | - | | -33.57 (42.84) | | -48.37  (-) | | 5.6 (14.3) | 5 (19) | | - |
|  |  |  |  |  |  | 36.14 (48.60) | |  |  |  |  |
| **SOL** (minutes) | -8.62 (53.76) | - | | - | | -1.99 (-) | | 2.5 (11.4) | 4 (9) | | - |
|  |  |  |  |  |  | 23.22 (24.47) | |  |  |  |  |
| **SE** (%) | -5.82 (17.67) | - | | 13.25 (-) | | 11.78 (-) | | -1.8 (4.5) | - | | 14.9 (-) |
|  |  |  |  |  |  | - | |  |  |  |  |
| **Time in N1+N2**  **(“light sleep”)**  (minutes) | -29.81 (67.98) | - | | - | | 138.30  (-) | | - | -34 (34) | | - |
|  |  |  |  |  |  | -68.82 (76.77) | |  |  |  |  |
| **Time in N3**  **(“deep sleep”)**  (minutes) | -14.64 (59.97) | - | | - | | -59.40  (-) | | - | 24 (28) | | - |
|  |  |  |  |  |  | 74.24 (46) | |  |  |  |  |
| **Time in REM** (minutes) | 19.49 (36.40) | - | | - | | 18.50  (-) | | - | 1 (27) | | - |
|  |  |  |  |  |  | -2.78 (33.32) | |  |  |  |  |
| **Awake** (minutes) | 28.36 (72.69) | - | | - | | -96.97  (-) | | - | - | | - |
|  |  |  |  |  |  | 41.93 (55.92) | |  |  |  |  |
|  | | | | | | | | | | | |
| **Outcomes Epoch by Epoch analysis** | | | | | | | | | | | |
|  | **Mi Band 5** | | **Alta HR** [36] | | **Charge 2** [37] | | **Õura Ring** [39] | | | **Actigraphy** [60] | |
| **Accuracy** | 0.78 (0.13) | | - | | - | | - | | | - | |
| **Sensitivity** | 0.89 (0.16) | | 0.87 (-) | | 0.96 (-) | | 0.95 (4.5) | | | 0.96 (-) | |
| **Specificity** | 0.35 (0.11) | | 0.44 (-) | | 0.61 (-) | | 0.48 (19.1) | | | 0.37 (-) | |
| **Cohen’s kappa** | 0.22 (0.23) | | - | | 0.52 (0.14) | | - | | | 0.79 (-) | |
