# Supplementary material for: Quality of Sleep Data Validation From the Xiaomi Mi Band 5 Against Polysomnography: Comparison Study
Source: J Med Internet Res. 2023 May 19;25:e42073. doi: 10.2196/42073 (PMC10238963; doi:10.2196/42073)
Supplement: Multimedia Appendix 2 [file jmir_v25i1e42073_app2.docx]

**Quality of Sleep Data Validation from the Xiaomi Mi Band 5 Compared With Polysomnography**

## Appendix 2

## Analysis of data according to the type of sleep disorder

The group with sleep disorders was divided according to the type of disorder to determine the performance of the Xiaomi Mi Band 5. The authors decided to divide the group between people with obstructive sleep apnea (OSA, n = 18) and people with other types of sleep disorders (“Other SDis”, n = 7) such as insomnia, narcoleptic syndrome, a combination of hypersomnia and narcoleptic syndrome, and a combination of sleep apnea syndrome and hypoventilation syndrome. The authors chose this division because the number of people with the other sleep problems is small.

### **Results**

### **Comparison of PSG and Xiaomi Mi Band 5 Sleep Measures**

Table S2 shows the results for PSG and the Xiaomi Mi Band 5 in OSA and “Other SDis” group There were no significant differences in sleep measures such as "initial sleep onset," TSPD, SE, and “light sleep” in the OSA group. However, the Xiaomi Mi Band 5 significantly overestimated PSG TST, PSG SOL, and PSG "deep sleep", and it underestimated PSG WASO, PSG awakenings, PSG REM, and PSG awake time. In the “Other SDis” group, there were no significant differences in WASO, "light sleep", and REM sleep variables. Nevertheless, the Xiaomi Mi Band 5 significantly overestimated PSG TSPD, PSG TST, PSG "deep sleep", and the percentage in PSG SE. This device underestimated PSG "initial sleep onset ", PSG awakenings and PSG awake time.

**Table S2.** Comparison of polysomnography (PSG) and Xiaomi Mi Band 5 sleep measures in the OSA and “Other SDis” groups.

|  | | | **PSG** | | **Xiaomi Mi Band 5** | | t | z | *P* | d |  |
| --- | --- | --- | --- | --- | --- | --- | --- | --- | --- | --- | --- |
|  |  |  | Mean (SD) | ±95% CI | Mean (SD) | ±95% CI |  |  |  |  |  |
| **Lights-on** (hh:mm) | *OSA* | | 07:02 (00:19) | 07:12-06:52 | - | - | - | - | - | - |  |
|  | *Other SDis* | | 23:49 (00:33) | 07:24-06:34 | - | - | - | - | - | - |  |
| **Lights-off** (hh:mm) | *OSA* | | 23:39 (00:32) | 23:55-23:23 | - | - | - | - | - | - |  |
|  | *Other SDis* | | 06:59 (00:27) | 00:20-23:18 | - | - | - | - | - | - |  |
| **Initial sleep onset** (hh:mm) | *OSA* | | 00:07 (00:45) | 00:29-23:44 | 00:16 (01:16) | 00:54-23:38 | -0.54 | - | **0.595** | -0.228* |  |
|  | *Other SDis* | | 01:02 (01:25) | 02:21-023:43 | 00:02 (01:09) | 01:06-22:59 | 2.32 | - | 0.059 | 0.878*** |  |
| **TIB**  (minutes) | *OSA* | | 445.90 (38.87) | 465.24-426.57 | - | - | - | - | - | - |  |
|  | *Other SDis* | | 428.80  (47.09) | 472.36-385.25 | - | - | - | - | - | - |  |
| **TSPD** (minutes) | *OSA* | | 412.96 (45.83) | 435.75-390.16 | 403.46 (49.79) | 428.23-378.70 | 0.91 | - | **0.378** | 0.213* |  |
|  | *Other SDis* | | 353.32  (89.53) | 436.12-270.52 | 400.53 (63.25) | 459.02-342.04 | -2.03 | - | 0.089 | -0.767** |  |
| **TST** (minutes) | *OSA* | | 353.67  (59.46) | 383.24-324.10 | 379.14 (56.93) | 407.45-350.82 | - | -1.87 | 0.031 | -0.440* |  |
|  | *Other SDis* | | 267.30  (115.24) | 373.88-160.72 | 349.70  (111.77) | 453.07-246.32 | - | -2.98 | 0.024 | -1.13*** |  |
| **WASO**  (minutes) | *OSA* | | 59.29  (48.77) | 83.54-35 | 24.32 (30.45) | 39.47-9.18 | - | 2.36 | 0.031 | 0.556** |  |
|  | *Other SDis* | | 86.03  (81.54) | 161.44-10.61 | 50.83 (83.63) | 128.18--26.52- | - | 1.03 | **0.341** | 0.391* |  |
| **Awakenings** (> 5 minutes) (number per night) | *OSA* | | 3.17 (1.73) | 4.02-2.30 | 0.77 (0.88) | 1.21-0.34 | - | 4.84 | <0.001 | 1*** |  |
|  | *Other SDis* | | 4.14 (3.24) | 7.13-1.15 | 0.57 (1.13) | 1.62--0.47 | - | 2.69 | 0.036 | 1*** |  |
| **SOL** (minutes) | *OSA* | | 22.87  (19.69) | 32.66-13.07 | 49.14 (55.50) | 76.74-21.55 | - | -1.81 | 0.087 | -0.427* |  |
|  | *Other SDis* | | 68.98  (68.45) | 132.29-5.67 | 21.77 (24.38) | 44.32--0.78 | - | 2.03 | 0.089 | 0.767** |  |
| **SE** (%) | *OSA* | | 81.12  (11.28) | 86.73-75.51 | 84.40 (13.53) | 91.13-77.67 | - | -0.80 | **0.433** | -0.289* |  |
|  | *Other SDis* | | 63.44  (27.47) | 88.85-38.03 | 82.17(23.41) | 103.83-60.51 | - | -3.10 | 0.021 | -1.17*** |  |
| **Time in N1**  (minutes) | *OSA* | | 10.86  (8.50) | 15.08-6.63 | - | - | - | - | - | - |  |
|  | *Other SDis* | | 13.86(9) | 22.18-5.53 | - | - | - | - | - | - |  |
| **Time in N2**  (minutes) | | *OSA* | 219.63 (47.63) | 243.32-195.95 | - | - | - | - | - | - | |
|  |  | *Other SDis* | 166.86 (81.17) | 241.94-91.80 | - | - | - | - | - | - | |
| **Time in N1+N2**  (“light sleep”) (minutes) | | *OSA* | 230.49 (44.66) | 252.70-208.29 | 237.22 (48.73) | 261.45-212.99 | -0.39 | - | **0.699** | -0.193* | |
|  |  | *Other SDis* | 180.73 (84.83) | 259.18-102.27 | 234.99  (85.62) | 314.18-155.80 | -1.93 | **-** | **0.101** | -0.731** | |
| **Time in N3**  (“deep sleep”) (minutes) | | *OSA* | 51.19 (29.98) | 66.10-36.28 | 81.56  (34) | 98.46-64.65 | -3.51 | **-** | 0.003 | -0.828*** | |
|  |  | *Other SDis* | 38.57 (26.61) | 63.18-13.95 | 63.69  (29.54) | 91.01-36.37 | -1.74 | - | **0.131** | -0.661** | |
| **Time in REM** (minutes) | | *OSA* | 71.98 (28.14) | 85.97-57.99 | 48.81(36.21) | 66.82-30.80 | 2.87 | - | 0.010 | 0.678** | |
|  |  | *Other SDis* | 48  (34.05) | 79.49-16.50 | 51.01 (37.85) | 86.01-16 | -0.35 | - | **0.749** | -0.227* | |
| **Awake** (minutes) | | *OSA* | 81.64  (50.46) | 106.73-56.54 | 56.17(48.22) | 80.15-32.19 | - | 1.86 | 0.079 | 0.440** | |
|  |  | *Other SDis* | 155 (114.71) | 261.11-48.89 | 72.60 (93.56) | 159.14-13.93 | - | 2.98 | 0.024 | 1*** | |
| REM, rapid-eye-movement; SDis, sleep disorder; SE, sleep efficiency; SOL, sleep onset latency; TIB, time in bed; TSPD, total sleep period duration; TST, total sleep time; WASO, wake after sleep onset.  t values correspond to t-tests. z values correspond to the Mann-Whitney-Wilcoxon test. d values correspond to Cohen’s d: small effect (*), moderate effect (**), or large effect (***). | | | | | | | | | | | |

### **Bland-Altman Plots**

Table S3 presents Bland Altman biases, SD ±95% CI, and the lower and upper agreement limits. In the OSA group, the Xiaomi Mi Band 5 significantly overestimated PSG TST by 25.47 minutes, PSG SOL by 26.27 minutes, and PSG “deep sleep” by 30.36 minutes. It underestimated PSG WASO by 34.96 minutes, PSG awakenings by 2.39 epochs, PSG REM sleep by 23.17 minutes, and PSG awake by 25.46 minutes. Furthermore, this device significantly overestimated PSG TST by 82.40 minutes, PSG TSPD by 47.21 minutes, PSG SE by 18.73 points, PSG “light sleep” by 54.27 minutes, and PSG “deep sleep” by 25.12 minutes in the “Other SDis”. It underestimated PSG “initial sleep onset” by 59 minutes, PSG awakenings by 3.57 epochs, and PSG awake by 82.40 minutes.

**Table S3.** Bland-Altman parameters for the comparison between polysomnography (PSG) and the Xiaomi Mi Band 5 in the OSA and “Other SDis” groups.

|  | | **Bias (SD)** | ±**95% CI of the bias** | **Lower agreement limit** | **Upper agreement limit** | **Number of participants exceeding the agreement limits** |
| --- | --- | --- | --- | --- | --- | --- |
| **Initial sleep onset** (hh:mm) | *OSA* | -00:09 (01:13) | 00:26-00:45 | -02:12 | 02:30 | 1 |
|  | *Other SDis* | 00:59 (01:07) | 02:02-00:03 | -01:12 | 03:10 | 3 |
| **TST** (minutes) | *OSA* | -25.47  (57.87) | 3.30-54.25 | -138.89 | 87.96 | 0 |
|  | *Other SDis* | -82.40 (72.95) | -14.93-149.87 | -225.39 | 60.58 | 1 |
| **TSPD** (minutes) | *OSA* | 9.49  (44.47) | 31.61-12.62 | -77.68 | 96.66 | 0 |
|  | *Other SDis* | -47.21 (61.52) | 9.69-104.10 | -167.78 | 73.36 | 1 |
| **WASO** (minutes) | *OSA* | 34.96 (62.90) | 66.24-3.68 | -88.32 | 158.25 | 0 |
|  | *Other SDis* | 35.19 (89.99) | 118.42-48.03 | -141.19 | 211.57 | 1 |
| **Awakenings**  (> 5 minutes) (number per night) | *OSA* | 2.39 (2.09) | 3.43-1.35 | -1.7 | 6.48 | 2 |
|  | *Other SDis* | 3.57 (3.50) | 6.81-0.33 | -3.18 | 10.32 | 1 |
| **SOL** (minutes) | *OSA* | -26.27 (61.47) | 4.29-56.84 | -146.76 | 94.21 | 1 |
|  | *Other SDis* | 47.21  (61.52) | 104.10-9.68 | -73.36 | 167.78 | 1 |
| **SE** (%) | *OSA* | -3.28  (17.31) | 5.33-11.88 | -37.21 | 30.66 | 1 |
|  | *Other SDis* | -18.73  (15.97) | -3.95-33.50 | -50.03 | 12.58 | 1 |
| **Time in N1+N2**  (“light sleep”) (minutes) | *OSA* | -6.72  (72.55) | 29.36-42.80 | -148.93 | 135.48 | 1 |
|  | *Other SDis* | -54.27  (74.28) | 28.07-122.97 | -199.87 | 91.33 | 1 |
| **Time in N3**  (“deep sleep”) (minutes) | *OSA* | -30.36  (36.66) | -12.13-48.59 | -102.21 | 41.48 | 0 |
|  | *Other SDis* | -25.12  (38.02) | 10.05-60.29 | -99.65 | 49.41 | 0 |
| **Time in REM** (minutes) | *OSA* | 23.17  (34.16) | 40.16-6.18 | -43.79 | 90.13 | 0 |
|  | *Other SDis* | -3.01  (23.77) | 18.97-24.99 | -49.60 | 43.57 | 0 |
| **Awake** (minutes) | *OSA* | 25.46  (57.87) | 54.25-3.31 | -87.96 | 138.90 |  |
|  | *Other SDis* | 82.40(72.95) | 149.87-14.93 | -60.59 | 225.39 |  |
| CI, confidence interval; REM, rapid-eye-movement; SDis, sleep disorders; SE, sleep efficiency; SOL, sleep onset latency; TSPD, total sleep period duration; TST, total sleep time; WASO, wake after sleep onset. | | | | | | |

### **Epoch by Epoch analysis**

Table S4 shows the accuracy and Kappa levels for the sleep/wake and sleep stages classifications by Xiaomi Mi Band 5. In the sleep/wake epochs identification, the accuracy level was 0.77 and 0.74 for OSA and “Other SDis” groups. The accuracy in the detection of sleep stages was low, with values between 0.42 and 0.48 for both groups. Concerning Kappa, the values were slight in the OSA group, and they were between fair and slight in the “Other SDis” group.

**Table S4.** Overall accuracy and kappa statistics for 2-way and 4-way epoch by epoch classification for OSA and “Other SDis” groups.

|  | **2-way** | | **4-way** | |
| --- | --- | --- | --- | --- |
|  | **Accuracy** | **Kappa** | **Accuracy** | **Kappa** |
|  | Mean (SD) | Mean (SD) | Mean (SD) | Mean (SD) |
| *OSA* | 0.77 (0.10) | 0.16 (0.23) | 0.42 (0.10) | 0.10 (0.14) |
| *Other SDis* | 0.74 (0.15) | 0.22 (0.33) | 0.48 (0.07) | 0.13 (0.15) |
| Cohen’s Kappa (k): 0-0.2 (slight); 0.21-0.40 (fair); 0.41-0.60 (moderate); 0.61-0.80 (substantial); > 0.80 (almost perfect) | | | | |

### **Conclusion**

In general, Xiaomi significantly overestimated and underestimated most of the sleep measures in both groups. However, the Xiaomi device was less accurate in the estimates of sleep variables in the "Other SDis" group. Concerning the performance of Xiaomi in the detection of sleep/wake stages and sleep stages, the results were similar in both groups. However, the Kappa coefficients were slightly higher in the "Other SDis" group.
